# PARP Inhibition Attenuates Acute Kidney Allograft Rejection by Suppressing Cell Death Pathways and Activating PI-3K-Akt Cascade

**DOI:** 10.1371/journal.pone.0081928

**Published:** 2013-12-03

**Authors:** Karoly Kalmar-Nagy, Peter Degrell, Aliz Szabo, Katalin Sumegi, Istvan Wittmann, Ferenc Gallyas, Balazs Sumegi

**Affiliations:** 1 Department of Surgery, University of Pecs Medical School, Pecs, Hungary; 2 2nd Department of Internal Medicine and Nephrology Centre, University of Pecs Medical School, Pecs, Hungary; 3 Department of Biochemistry and Medical Chemistry, University of Pecs Medical School, Pecs, Hungary; 4 Szentagothai Research Center, University of Pecs, Pecs, Hungary; Pennington Biomedical Research Center, United States of America

## Abstract

**Background:**

Novel immunosuppressive therapy facilitates long term allograft survival, but acute tubular necrosis and ischemia-reperfusion during transplantation can compromise allograft function. These processes are related to oxidative stress which activates poly- (ADP-ribose) polymerase (PARP) contributing to the activation of cell death pathways. Here we raised the possibility that PARP inhibition curbs cell death pathways and shifts kinase signaling to improved graft survival.

**Methods Findings:**

In an acute rat kidney rejection model, we provided evidence that the PARP inhibitor 4-hydroxy-quinazoline (4OHQ) attenuates rejection processes initiated oxidative/nitrosative stress, nuclear poly-ADP-ribosylation and the disintegration of the tubulo-interstitial structures. The PARP inhibitor attenuated rejection processes induced pro-apoptotic pathways by increasing Bcl-2/Bax ratio and suppressing pro-apoptotic t-Bid levels. In transplanted kidneys, the cell death inducing JNK1/2 is normally activated, but PARP inhibition suppressed this activation with having only modest effects on ERK1/2 and p38 MAP kinases. In untreated transplanted kidneys, no significant alterations were detected in the cytoprotective PI-3K-Akt pathway, but the PARP inhibitor significantly activated Akt (by S473 phosphorylation) and suppressed GSK-3β, as well as activated acute NF-kappaB activation contributing to graft protection.

**Conclusion:**

These data show the protective role of PARP inhibition on graft survival by attenuating poly-ADP-ribosylation, oxidative stress, suppressing pro-apoptotic and increasing anti-apoptotic protein level, and by shifting MAP kinases and PI-3-K-Akt pathways to cytoprotective direction. Thus, addition of PARP inhibitors to standard immunosuppressive therapies during kidney transplantation may provide increased protection to prolong graft survival.

## Introduction

Kidney transplantation is the best choice for patients with end-stage kidney disease. Due to cellular and humoral immune response, acute kidney damage however may be an important cause for graft loss [1]. Rejection is often characterized and mediated by the presence of at least 4 types of committed helper T cells (T helper (Th)1, Th2, Th17, and regulatory T cells) in the interstitial, tubular, and glomerular compartments [1], [2]. The presence of these cells is often associated with vasculitis, deposition of immunoglobulins in peritubular capillaries [3]. An activation of the complement cascade [4] and the presence of proinflammatory cytokines (e.g. TNF-α and IL-17) may also be involved. Anti-inflammatory cytokines, such as TGF-β, the transcription factor of regulatory T cells and FoxP3 on the other hand facilitate better transplant survival [3]. Allograft damage may be also caused by leukocyte infiltration, recruitment of neutrophils and monocytes on activated endothelial cells contributing to tubular interstitial inflammation and oxidative stress. These processes lead to cell death and chronic dysfunction [5]. Other types of injuries, such as ischaemia-reperfusion, acute rejection and hyperacute rejection are related to inflammation and oxidative stress affecting the outcome of transplantation [4]. Earlier data demonstrated that higher oxidative stress markers in the serum of transplanted patients generally result in less functional kidney indicating the significance of oxidative stress in the decline of graft function [6].

It is known, that components of standard immunosuppressive therapy (e.g. Cyclosporine A and Tacrolimus) cause oxidative stress and activates MAPK signaling which lead to glomerular dysfunction and subsequent nephrotoxicity [7], [8]. Therefore, a therapy to protect transplanted kidney tissues from oxidative stress and oxidative stress related processes in addition to attenuation of rejection processes by immunosuppressive therapy may have clinical significance. Efforts to activate cytoprotective pathways using carbamylated erythropoietin [9] or to possess antioxidant activity via liposomal curcumin [10] support our hypothesis.

Poly- (ADP-ribose) polymerase (PARP)-1 is a high copy number nuclear enzyme which is activated by DNA-breaks and catalyzes the poly-ADP-ribosylation of nuclear proteins utilizing NAD^+^ [11], [12]. Oxidative stress via the induction of DNA breaks can activate PARP leading to NAD^+^ and ATP depletion followed by necrotic cell death [13]. In addition, PARP activation through the destabilization of mitochondrial outer membranes promotes the release and nuclear translocation of Apoptosis-Inducing Factor (AIF) and Endonuclease G leading to apoptosis [14], [15]. Therefore, PARP inhibitors can be used to prevent oxidative stress induced cell death [13]–[15]. Oxidative stress induced activation of PARP promotes JNK and p38 MAPK activation while PARP inhibitors suppresses their activation [16]–[18]. We found that inhibition of PARP in oxidative stress activates the expression of MAP kinase phosphosphatase-1 (MKP-1/Dusp1) which is the major phosphatase, which dephosphorylates and inactivates the MAP kinases [19]. From these data we can conclude that PARP inhibitors have the potential to protect different tissues from oxidative stress [11], [20], [21], and can regulate a favorable way MAP kinases [19] and inflammatory processes [22].

Therefore, PARP inhibitors have protective effects in various oxidative stress related disease-models by preventing compromised energy status and by preventing other cell death promoting effects of PARP activation [11, 12, 23, 24). Excessive activation of PARP by stress stimuli, such as reactive oxygen species (ROS) formation has been associated with the pathogenesis of various diseases, including cerebral ischemia, Parkinson’s disease [25], [26], ischemia-reperfusion (IR) - induced cardiac dysfunction [27], [28], development of diabetic complications [29] and angiogenesis [30].

Studying the renal graft dysfunction in acute rat rejection model we found PARP inhibitor 4-hydroxyquinazolone (4OHQ) has protective effects. It prevents disintegration of the tubulointerstitial structures, decreases oxidative stress markers, increases anti-apoptotic Bcl-2 levels, suppresses cell death by inducing JNK activation and activates the cytoprotective PI-3K-Akt pathway. Our data suggest adding PARP inhibitors to immunosuppressant regiments during kidney transplantation may be advantageous in the acute rejection period to protect the graft against ischemia-reperfusion and other types of oxidative stress induced damages.

## Materials and Methods

### Materials

Protease inhibitor cocktail and all chemicals for cell culture were purchased from Sigma Aldrich Co. (Budapest, Hungary). PARP inhibitor 4-hydroxiquinazoline was purchased from Sigma-Aldrich as previously [31], [32]. All reagents were of the highest purity commercially available.

### Animals and Renal Transplantation

Inbred male rats from Charles River Laboratories GmbH (Hungary) were used for all transplant experiments. Fisher 344 rats; body weight (bw) 230 to 250 g served as donors for kidney transplants. Lewis rats (LEW RT1l; bw, 200 to 230 g) served as recipients. Ureters were directly inserted into the bladder. All animals were fed with standard rat chow and had free access to tap water. All experiments conformed to the Guide for the Care and Use of Laboratory Animals published by the US National Institutes of Health (NIH Publication No. 85-23, revised 1996), and was approved by the Animal Research Review Committee of the University of Pecs Medical School.

Renal transplantation was performed by a technique modified from [33]. Animals were anaesthetized with i.p. ketamine hydrochloride. The aorta of the donor animal was cannulated and the abdominal organs were perfused in situ with 10 ml of hyperosmotic citrate kidney perfusion solution (Soltran®, Baxter). Right kidney was removed and deep frozen as control. Left kidney was removed with a long ureter. The recipient operation was performed via median laparotomy approach. The artery of the graft was anastomosed end-to-side to the infrarenal aorta using 9-0 interrupted suture (Prolene®, Ethicon). The renal vein of the graft was anastomosed using the same technique to the infrarenal vena cava inferior. The ureter was directly inserted into the bladder. The recipient animal’s native kidneys were left intact. The cold ischemia time varied between 30 and 50 minutes. The anastomosis time was between 40 and 50 minutes in every group.

### Experimental Protocol and Experimental Groups

4-hydroxyquinazoline (Sigma) was dissolved in water diluted to 0.9% sodium chloride and injected subcutaneously twice a day at a dose of 20 mg/kg bw in all treated animals. The experimental groups were as follows: group 1: Fisher 344 rats injected with 0.9% sodium chloride. 2. Fisher 344 rats injected with 20 mg/kg bw 4-hydroxyquinazoline in 0.9% sodium chloride. 3. Fisher 344 kidneys into LEW rats, injected with 0.9% sodium chloride. 4. Fisher 344 kidneys into LEW rats injected with 20 mg/kg bw 4-hydroxyquinazoline in 0.9% sodium chloride. After 10 days rats were sacrificed by an overdose of i.p. ketamine hydrochloride, kidneys were removed and subjected to morphological and biochemical analysis.

### Renal histology

Renal allografts were removed in deep anesthesia, quickly blotted free of blood, weighed, and processed as required for histology and immunohistology. Kidneys were fixed in 10% formalin, embedded into paraffin and 5 µm thin sections were cut with microtome. Sections were stained with hematoxylin–eosin and digital photos were taken. Haematoxylin-eosin staning and periodic acid Schiff (PAS) reaction was also performed.

### Immunohistochemical staining

Slides were deparaffinized in xilene, rehydrated in graded ethanol series, and washed in distilled water. Heat induced epitope retrieval was performed by boiling the tissue sections in citrate buffer (HISTOLS® Citrate Buffer, cat# 30010; Histopathology Ltd.) in a microwave oven at 750 W followed by cooling at room temperature for 20 minutes. Slides were washed in Tris buffered saline (TBS) solution (pH  =  7,6) followed by blocking of endogenous peroxidase (Peroxidase blocking, cat#30012, Histopathology Ltd.) for 10 minutes at room temperature. Slides were washed in TBS. Nonspecific sites were blocked (Background Blocking Protein Solution, cat#30013, Histopathology Ltd.) for 10 minutes at room temperature. Without washing, the following primary antibodies were applied: anti-bax (Lab Vision/Thermo Fischer Scientific, cat# RB-9206, in 1:200 dilution), anti-bcl-2 (Lab Vision/Thermo Fischer Scientific, cat# MS-123, in 1∶100 dilution), anti-Nitrotyrosine (Milliopre, cat#AB5411 in 1∶500 dilution). Incubation with the primary antibodies was performed for 1 hour at room temperature followed by washing in TBS. Secondary antibody (HISTOLS® -R Detection System, anti-rabbit, cat# 30011.R; and HISTOLS® -M Detection System, anti-mouse, cat# 30011.M Histopathology Ltd.) was applied for 30 minutes at room temperature followed by repeated washing in TBS. Sections were incubated with 3-amino-9-ethylcarbazol (HISTOLS® -Resistant AEC Chromogen/Subsrtate System, cat# 30015.K, Histopathology Ltd.) or 3,3’-Diaminobenzidine (HISTOLS® DAB Chromogen/Subsrtate System, cat#30014.K, Histopathology Ltd.), washed in distilled water, counterstained with haematoxylin followed by incubation in tap water. Negative control was incubated with antibody diluent instead of the primary antibody and applying anti-rabbit or anti-mouse secondary antibody. Sections were then dehydrated, cleared in xylene and mounted with permanent mounting medium.

### Morphometric analysis

Adobe Photoshop program was used to mark the positive area in the immunohistochemistry manually, then converted to black, while the remaining area to white. For each image, the “area of interest” as well as the total image area were highlighted, and measured using Scion Image program. Finally, the ratio of „area of interest”/total image size was calculated and expressed as percentage. Comparison of means of the treatment groups was carried out using ANOVA with Bonferroni’s post-hoc test. A value of p<0.05 was considered as statistically significant.

### Immunoblotting

Kidney samples were homogenized in ice-cold isotonic Tris buffer (50 mM, pH 8.0) containing 0.5 mM sodium metavanadate, 1 mM EDTA, and a protease inhibitor cocktail (1∶1000; Sigma–Aldrich) as described previously [19]. Proteins and phosphorylation sites were determined from the tissue homogenates after sonication. Proteins were precipitated by trichloroacetate, washed three times with −20 °C acetone, dissolved in Laemmli sample buffer, separated on 12% SDS–polyacrylamide gels, and transferred to nitrocellulose membranes. After being blocked (2 h with 3% nonfat milk in Tris-buffered saline), the membranes were probed overnight at 4 °C with antibodies recognizing the following antigens: phospho-JNK (T183/Y185), phospho-p38 MAP kinase (Thr180/Tyr182), phosphor-ERK1/2 (Thr202/Tyr204), phosphor-Akt (S473), Akt, phosphor-GSK-3beta (Ser9), Bcl-2and Bax (Cell Signaling Technology), t-Bid (Santa Cruz), actin (Sigma), NF-kappaB, phospho-NF-kappaB (Ser536) (Cell Signaling) Primary antibodies were used in 1∶1000 dilution. For nuclear NF-kappaB determination kidneys were disrupted and the nuclei were separated as described before [29].

Anti-poly-ADP-ribose monoclonal antibody was a gift from Laszlo Virag (Debrecen, Hungary). The membranes were washed six times for 5 min in Tris-buffered saline (pH 7.5) containing 0.2% Tween before addition of goat anti-rabbit horseradish peroxidase-conjugated secondary antibody (1∶3000 dilution; Bio-Rad). The protein bands were visualized with enhanced chemiluminescence labeling using an ECL immunoblotting detection system (Amersham Biosciences). Developed films were scanned and the pixel volumes of the bands were determined using the NIH ImageJ software, with the values in ratios of intensity. Each experiment was repeated a minimum of three times.

Kidney lysates from 20 mg tissues were prepared and incubated with 100 µL of magnetic beads bound Histon H1 antibody. Samples were incubated overnight at 4°C with gentle mixing, and magnetic beads were washed three times with TBS–Tween 20 (0.05%) prior to the addition of 30 µL sample buffer. Histon H1 content and poly-ADP-ribosylation levels were determined by immunoblotting.

### Statistical analysis of immunoblot data

Five animals were allocated to each groups. All data were expressed as means±SEM from at least triplicate determinations.

ANOVA with a post hoc correction was used to determine differences. The Student t test was used to compare the mean values from the two groups. Differences were regarded as significant when the P value was <0.05.

## Results

### Histopathology of transplanted kidneys

**Figure 1:**
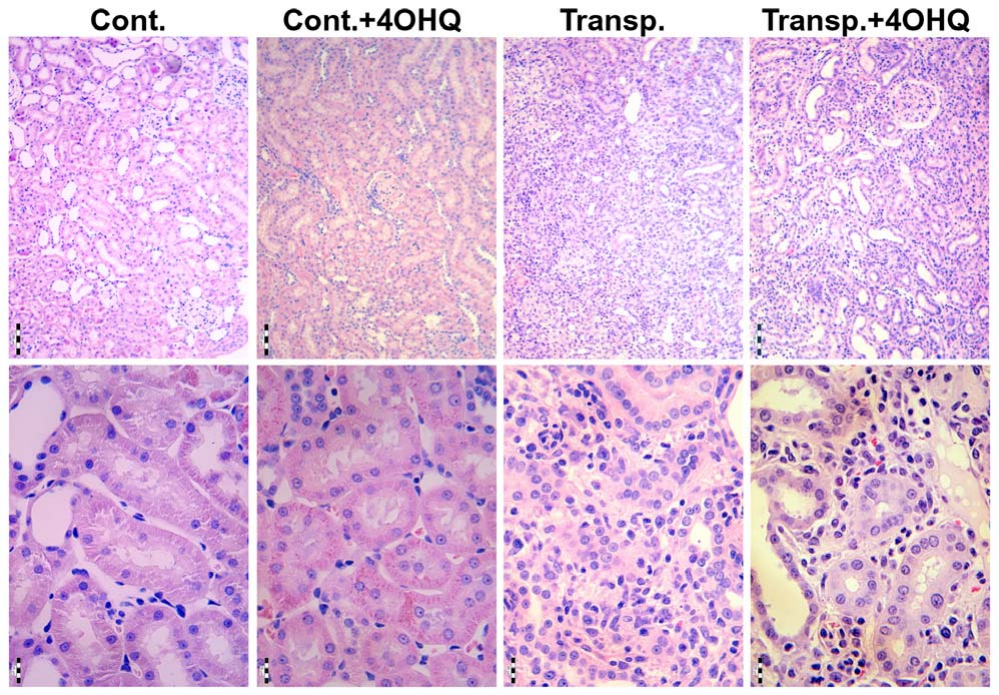
Effect of PARP inhibitor on the structure of tubulo-interstitial system of transplanted kidneys. Kidneys were fixed in 10% formalin, embedded into paraffin and 5 µm thin sections were cut with microtome. Sections were stained with hematoxylin–eosin (HE). Two representative images of different magnifications are presented for untreated unoperated (Cont.), 4OHQ treated unoperated (Cont.+4OHQ), untreated transplanted (Transp.), and 4OHQ treated transplanted kidneys.Scale bar: 100 and 20 µm for upper and lower row, respectively.

As it is demonstrated by HE and PAS slides, signs of vascular and cellular-interstitial rejection were seen in the untreated transplanted as well as in the PARP-treated transplanted rats, but not in the other two groups of experimental animals indicating that PARP inhibitor does not suppress the immune system. There were no significant histopathological alterations in the renal specimens of the control and the PARP-treated, non-transplanted rats. The transplanted kidneys showed disintegration of the tubulointerstitial structures as indicated by HE as well as PAS slides. On the contrary, less affected structures also in the tubulointerstitial region were observed in the PARP-treated transplanted animals (Fig. 1). These data indicate a protective role of PARP inhibition in the tubulointerstitial region.

### Effect of PARP inhibition on poly-ADP-ribosylation in transplanted kidneys

**Figure 2:**
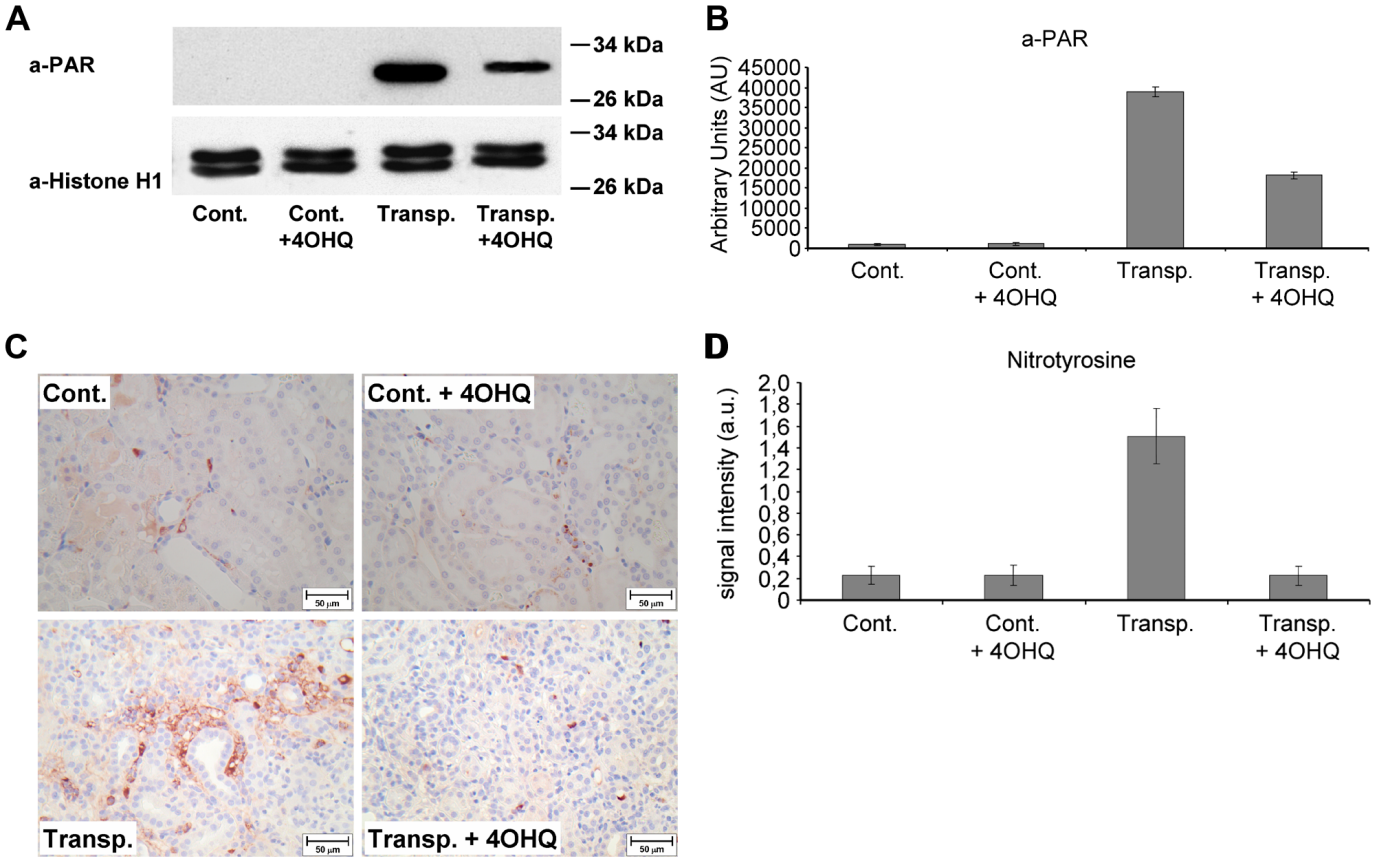
Effect of PARP inhibitor on poly-ADP-ribosylation (PAR) and protein nitration in transplanted kidneys. (A) Immunoblot analysis of poly-ADP-ribosylated HistonH1. HistonH1 was immunoprecipitated with anti-histonH1 antibody, dissolved in sample buffer, subjected to electrophoresis and blotting. Poly-ADP-ribose was detected by anti-PAR antibody. (B) Quantitative analysis of immunoblot samples (C) Representative images of nitrotyrosine immunohistochemistry. Brown color indicates nitrotyrosine positivity; scale bar: 50 µm. (D) Quantitative analysis of immunoblot samples. *: p<0,0001 untreated transplanted kidneys compared to others. ANOVA, Bonferroni post hoc test. Mean±SD. All details described under Materials and Methods.

It is well known that PARP poly-ADP-ribosylates histones [34]. Thus, to investigate the role of PARP during organ transplantation we studied histone poly-ADP-ribosylation in transplanted kidneys. Our results reveal that, ADP-ribosylation is highly detectable in transplanted kidneys when compared to controls (Fig. 2A, B). Comparing to the high signal intensity in the transplanted untreated kidneys PARP inhibition significantly decreased the ADP-ribosylation of histonH1 (Fig. 2A, B).

### Effect of PARP inhibition on the protein nitration in transplanted kidneys

In untreated control and PARP inhibitor treated control kidneys, no significant tyrosine nitrations were observed (Fig. 2C, D). However, marked nitrotyrosine positivity was detected in the transplanted untreated kidneys indicating that ischemia-reperfusion during transplantation followed by immunological rejection cause significant oxidative stress and nitration (Fig. 2C, D). In the transplanted PARP-treated kidneys, nitrotyrosine positivity was significantly weaker (Fig. 2C, D) suggesting that PARP inhibition decreased oxidative/nitrosative stress in transplanted kidneys.

### Effect of PARP inhibition on pro- and anti-apoptotic Bcl-2 analogues

**Figure 3:**
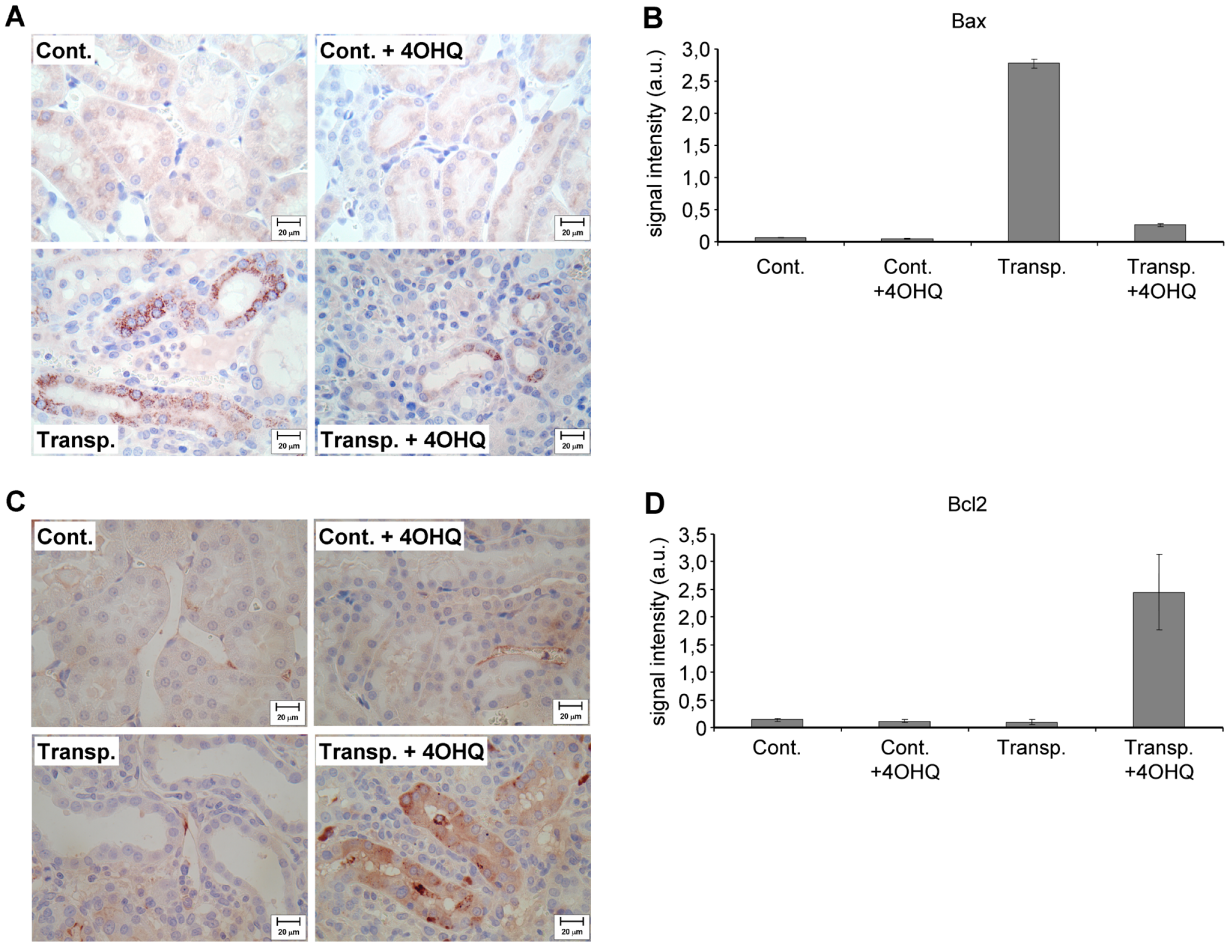
Effect of PARP inhibitor on the Bax and Bcl-2 immunohistology in transplanted and control kidneys. (A) All images show Bax immunohistochemistry, brown color indicates Bax positivity, scale bar: 20 µm. (B). Quantitative analysis of Bax immunohistochemistry samples *: p<0.0001 untreated transplanted kidneys compared to others. ; **: p<0.0001 PARP inhibitor treated transplanted kidneys compared to others. ANOVA, Bonferroni post hoc test. Mean±SD. (C) All images show Bcl-2 immunohistochemistry, brown color indicates Bcl-2 positivity, scale bar: 20 µm. All details described under Materials and Methods. (D) Quantitative analysis of the Bcl-2 immunohistochemistry. *: p<0,0001 transplanted and PARP-treated kidneys compared to others. ANOVA, Bonferroni post hoc test. Mean±SD. All details described under Materials and Methods.

Control kidneys with or without PARP inhibitor showed only insignificant Bax staining (Fig. 3A, B). In transplanted -untreated- kidneys, a strong, granular Bax positivity was observed in the tubular epithelial cells**.** Our observations are not surprising, since several works suggest that oxidative stress facilitates Bax expression [35], [36]. Inhibition of PARP in transplanted kidneys significantly suppressed Bax expression in the tubulointerstitial region (Fig. 3A, B), which is most likely related to decreased oxidative stress, or altered signaling of PARP inhibition [19]. Slight circumscribed immunostaining of BCL-2 in the peritubular capillary endothelial as well as in some tubular epithelial cells were seen in untreated control kidneys, PARP inhibitor treated control kidneys and in the transplanted untreated kidneys (Fig. 3C, D). Strong Bcl-2 positivity was present in the tubular epithelial cells of PARP-treated transplanted kidneys. That is, PARP inhibition significantly increased the Bcl-2/Bax ratio shifting the cells fate from apoptosis to survival.

**Figure 4:**
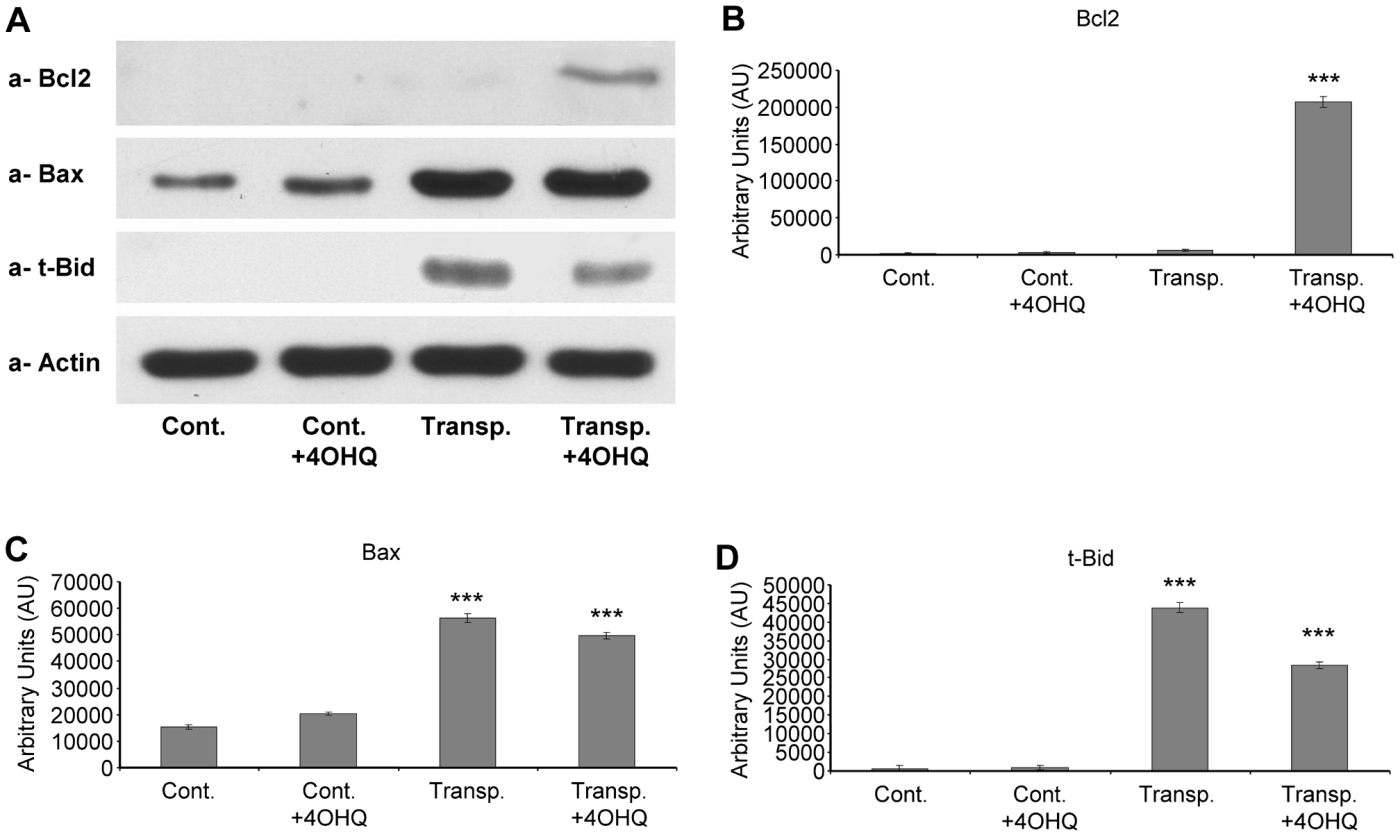
Effect of PARP inhibitor on Bcl-2, Bax and t-Bid protein levels in transplanted and control kidney samples. (**A**) Effects of PARP inhibitor on the Bcl-2, Bax and t-Bid protein levels in control and transplanted kidneys determined using immunoblotting with protein-specific primary antibodies. Actin was used as loading control. Representative blots of at least three parallel experiments are presented. (**B**) The bar diagrams represent pixel volumes of Bcl-2 bands. The bands were normalized to the appropriate actin band. *p<0.001 transplanted PARP inhibitor treated samples compared to other samples. The vertical axis represents pixel volume means±SEM of the scanned bands on the immunoblots in arbitrary units. The bar diagrams represent pixel volumes of Bax bands. The bands were normalized to the appropriate actin band. *p<0.01 transplanted PARP inhibitor treated samples, or transplanted untreated samples compared to control untreated and control PARP inhibitor treated samples. ** p<0.05 transplanted PARP inhibitor treated samples compared to transplanted untreated samples. The bar diagrams represent pixel volumes of t-Bid bands. The bands were normalized to the appropriate actin band. *p<0.01 transplanted PARP inhibitor treated samples, or transplanted untreated samples compared to control untreated and control PARP inhibitor treated samples. ** p<0.05 transplanted PARP inhibitor treated samples compared to transplanted untreated samples.

The results from total kidney homogenates support those from the immunohistology, namely, Bax increases in transplanted kidneys and Bcl-2 in the PARP inhibitor-treated transplanted kidneys (Fig. 4A, B). Therefore, PARP inhibition shifts Bcl-2/Bax ratio to Bcl-2 direction facilitating cell survival. We also investigated the expression of t-Bid, a molecule absent in control kidneys (treated or untreated by PARP inhibitor) (Fig. 4A, B). In the transplanted kidneys without PARP inhibitor treatment, t-Bid was very high, while PARP inhibition in transplanted kidneys decreased t-Bid level significantly (Fig. 4A, B). These data indicate PARP inhibition protects transplanted kidneys by shifting Bcl-2/Bax and by suppressing t-Bid.

### Effect of PARP inhibition on cytoprotective PI-3-K-Akt pathway and NF-kappaB activity

**Figure 5:**
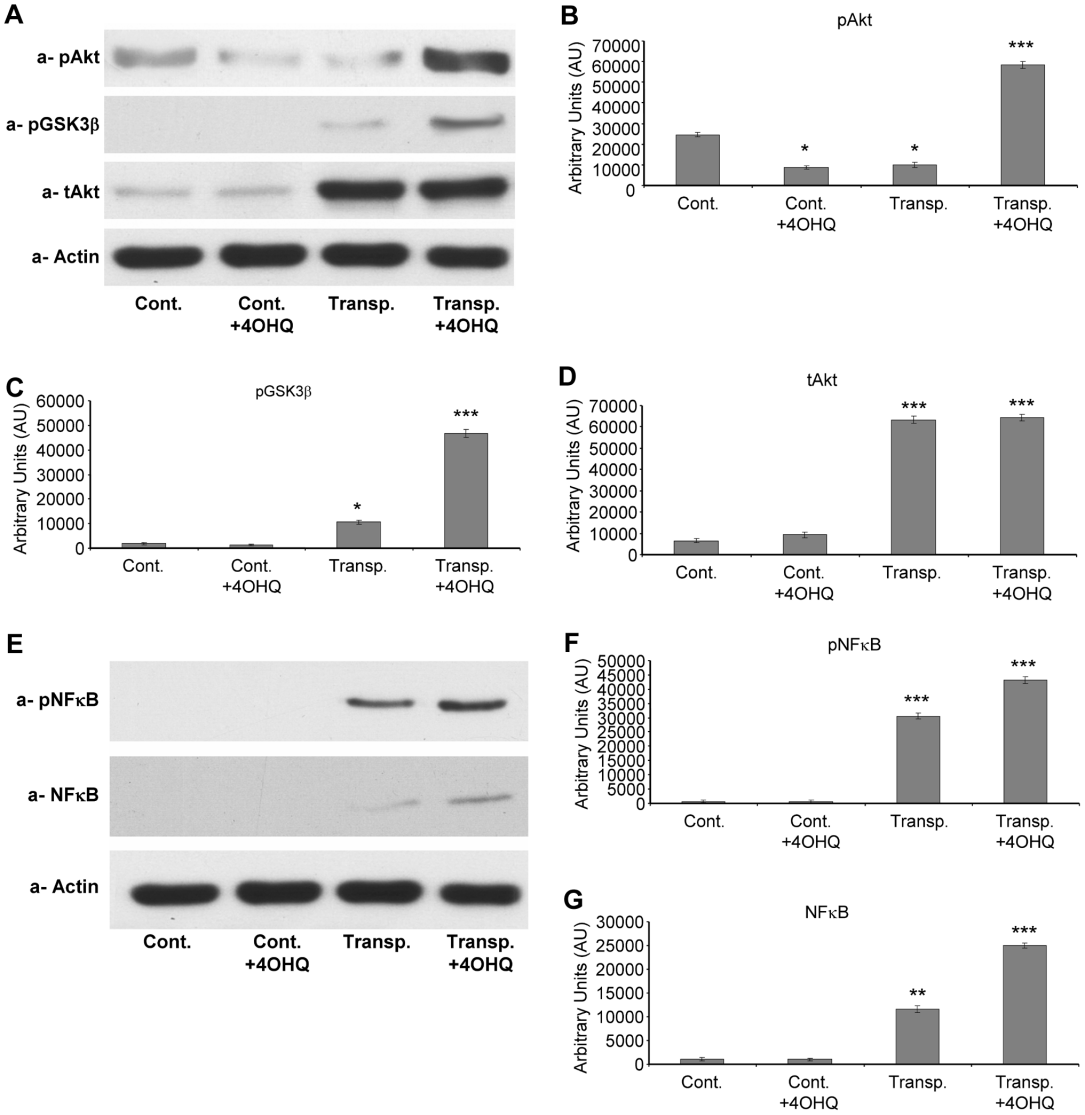
Effect of PARP inhibitor on the PI-3-kinase – Akt pathway and Akt level in transplanted and control kidney samples. (**A**) Effects of PARP inhibitor on the Akt1 protein, and phosphorylation of Akt1 and GSK-3 β in control and transplanted kidneys determined using immunoblotting with protein and phospho-specific primary antibodies. Actin was used as loading control. Representative blots of at least three parallel experiments are presented. **B**. The bar diagrams represent pixel volumes of phosphorylated Akt (serine 473) in kidney samples. The bands were normalized to the appropriate actin band. *p<0.01 transplanted PARP inhibitor treated samples compared to other samples. Difference between control samples (independently of PARP treatment) and transplanted untreated kidney samples were not significant. The bar diagrams represent pixel volumes of GSK-3β (serine 9) phosphorylation bands. The bands were normalized to the appropriate actin band. *p<0.001 transplanted PARP inhibitor treated samples compared to control PARP inhibitor treated, or untreated samples. **p<0.05 untreated transplanted kidney samples compared to control samples (independently of PARP treatment. *** p<0.01 transplanted PARP inhibitor treated samples compared untreated transplanted samples. The bar diagrams represent pixel volumes of Akt1 protein bands. The bands were normalized to the appropriate actin band. *p<0.001 transplanted kidney samples (independently of PARP inhibitor treatment) compared to control kidney samples (independently of PARP inhibitor treatment). (**C**) Effects of PARP inhibitor on nuclear NF-kappaB and p-NF-kappaB leveles in control and transplanted kidneys determined by immunoblotting with protein and phospho-specific primary antibodies. Actin was used as loading control. Representative blots of at least three parallel experiments are presented. (**D**) The bar diagrams represent pixel volumes of nuclear NF-kappaB protein bands. The bands were normalized to the appropriate actin band. *p<0.01 transplanted PARP inhibitor treated, or untreated, samples compared to control PARP inhibitor treated, or untreated control samples. **p<0.05 untreated transplanted kidney samples compared to PARP inhibitor treated transplanted kidney samples. The bar diagrams represent pixel volumes of nuclear p-NF-kappaB bands. The bands were normalized to the appropriate actin band. *p<0.001 transplanted PARP inhibitor treated, or untreated, samples compared to control PARP inhibitor treated, or untreated control samples. **p<0.05 untreated transplanted kidney samples compared to PARP inhibitor treated transplanted kidney samples. The vertical axes represent pixel volume means±SEM of the scanned bands of the immunoblots in arbitrary units.

In transplanted kidneys, the rejection processes are related to oxidative stress, which influences the activation of PI-3K-Akt pathway. Our data suggest (Fig. 5A, B) that transplantation significantly increases the quantity of total Akt-1 when compared to controls independently of PARP inhibitor treatment. However, the level of activated p-Akt-ser473 was significantly increased after PARP inhibition in transplanted kidneys when compared to untreated transplanted kidneys (Fig. 5A, B). GSK-3beta is a well known downstream target of Akt. Akt inactivates GSK-3beta by phosphorylation at serine 9, preventing GSK-3beta induced mitochondrial permeability transition and cell death [37]. Our data indicate PARP inhibitor inactivates GSK-3beta by phosphorylation at serine 9 in transplanted kidneys (Fig. 5A, B), suppressing GSK-3beta / mitochondria mediated cell death pathway. Next, nuclear NF-kappaB quantity and phosphorylation were determined (Fig. 5C, D). While the quantities in control kidneys were undetectable, transplanted samples contained increased levels of the protein. PARP inhibitor treatment further increased NF-kappaB levels when compared to untreated transplanted kidney samples (Fig. 5C, D). We found activating phosphorylation of NF-kappaB at Ser 536 was altered similarly. In control samples p-NF-kappaB were undetectable, in transplanted kidneys it was higher. PARP inhibitor treatment further increased p-NF-kappaB levels in the nuclei of transplanted kidneys.

### Effect of PARP inhibition on MAP kinases in transplanted kidneys

**Figure 6:**
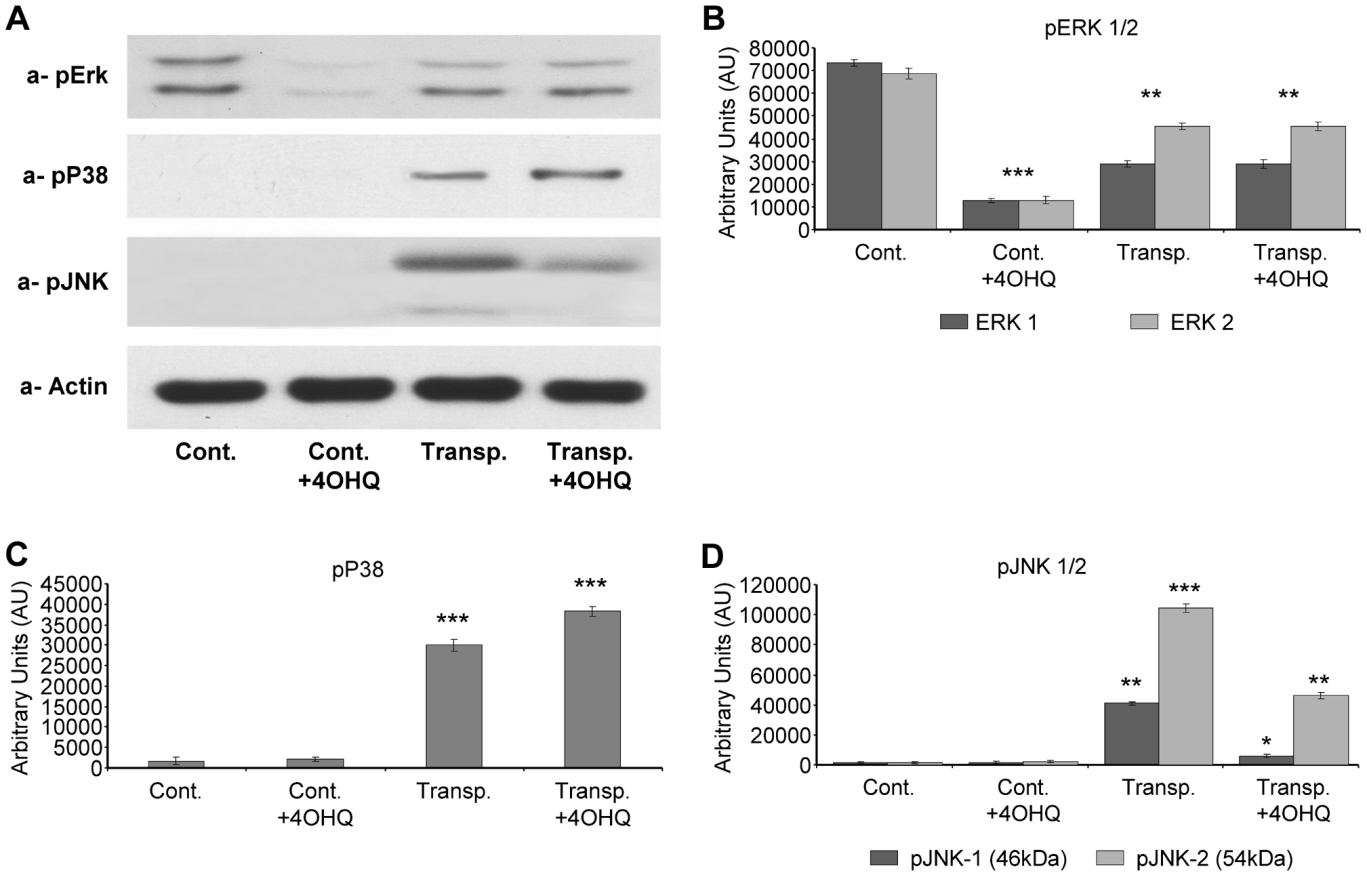
Effect of PARP inhibitor on activation and phosphorylation of ERK1/2, JNK1/2 and p38 MAP kinase pathways in transplanted and control kidney samples. (A) Effects of PARP inhibitor on the ERK1/2, JNK1/2 and p38 MAP kinase phosphorylation and activation in control and transplanted kidneys determined by immunoblotting with phospho-specific primary antibodies. Actin was used as loading control (B) The bar diagrams represent pixel volumes of ERK1/2 phosphorylation bands. The bands were normalized to the appropriate actin band. (p-ERK1) *p<0.001 control PARP inhibitor treated samples compared to control samples. **p<0.01 control PARP inhibitor treated samples compared to transplanted kidney samples independently from PARP inhibitor treatment. ***p<0.05 control untreated sample compared to transplanted kidney samples independently from PARP inhibitor treatment. (p-ERK2) *p<0.001 control PARP inhibitor treated samples compared to control samples. **p<0.01 control PARP inhibitor treated samples compared to transplanted kidney samples independently from PARP inhibitor treatment. ***p<0.05 control untreated sample compared to transplanted kidney samples independently from PARP inhibitor treatment. The bar diagrams represent pixel volumes of p-p38 MAK kinase phosphorylation bands. The bands were normalized to the appropriate actin band. *p<0.001 control samples compared to transplanted kidney samples (independently of PARP inhibitor treatment). **p<0.05 untreated transplanted kidney samples compared to control PARP inhibitor treated transplanted kidney samples. The bar diagrams represent pixel volumes of phosphorylated JNK1/2 bands. The bands were normalized to the appropriate actin band. (p-JNK1) *p<0.05 transplanted untreated kidney samples compared to all others samples. (p-JNK2) **p<0.001 control kidney samples (independently of PARP inhibitor treatment) comparing to transplanted samples. ***p<0.05 transplanted untreated kidney samples compared to transplanted PARP inhibitor treated kidney samples. The vertical axes represent pixel volume means±SEM of the scanned bands of the immunoblots in arbitrary units.

We analyzed three branches of the MAP kinases; ERK, JNK and p38. In the control organs, PARP inhibitor caused a decrease in ERK1/2 phosphorylation (Fig. 6A, B), while in the transplanted kidneys PARP inhibitor did not affect significantly ERK1/2 activation. While activation/phosphotylation of p38 MAP kinase in control hearts was undetectable, its phosphorylation increased upon transplantation. PARP inhibition further increased its activation (Fig. 5A, B). JNK phosphorylation was undetectable in control kidneys while JNK1/2 was significantly increased in transplanted kidneys. PARP inhibition suppressed their phosphorylation and activation (Fig. 6A, B). This effect of PARP inhibitors on the molecule might have mechanistic significance since JNK1 activation is capable of mitochondrial membrane destabilization and induction of cell death [38], [39].

## Discussion

Oxidative stress plays a significant role both in acute and chronic phases of kidney rejection. Causes may be ischemia-reperfusion induced oxidative stress, inflammatory cytokines or monocyte/macrophage produced reactive oxygen species [3], [6], [40]. The critical role of oxidative stress in rejection is supported by the observations that N-acetylcysteine prevents early rejection in animal models [41]. In addition, numerous human data indicate N-acetylcysteine improves graft function in transplanted patients [42]–[44]. Popular immune-suppressive drugs (tacrolimus, cyclosporine A) induce oxidative stress in long-term treatment contributing to the death of nephrons compromising long-term graft injury [7], [8]. Therefore, molecules that prevent oxidative stress induced cell damages may be important to preserve graft function.

It is well documented that oxidative stress activates PARP-1 by inducing DNA damage and single stranded DNA break formation [16], [45]. This activation may affect unfavorable signaling pathways (activation of JNK and p38 MAP kinase and suppression of Akt activation) that leads to cell death [34], [46]. Furthermore, PARP activation destabilizes the mitochondrial membrane systems [14], [18] resulting in release of pro-apoptotic proteins [47], [48]. Although PARP inhibitors were applied before during heart transplantation [49]–[51], there is no evidence about their effect on the kidney graft survival. Thus, we assumed PARP inhibition might be advantageous for kidney graft survival by suppressing cell death pathways and affecting kinase cascades in a favorable way.

We used the water soluble PARP inhibitor, 4OHQ as previously [16], [32]. Prolonged suppression of PARP activity by impeding DNA repair could be deleterious as it was shown in anti-cancer studies [52]. On the other hand, inhibiting oxidative stress induced overactivation rather than physiological activity of the enzyme did not result in significant pathological alteration even during very long exposures [16], [21], [32]. To this day, specificity of PARP inhibitors is elusive. Most likely, nobody tested any PARP inhibitor on all members of the PARP family. However, PARP-1 is considered to be responsible for about 90% of poly-ADP-ribosylation in mammalian cells [53]. Furthermore, we and others found that various PARP inhibitors and PARP-1 specific non-pharmacological inhibition of the enzyme such as PARP-1 siRNA or overexpression of PARP-1 DNA binding domain resulted in basically identical effects in different sytems [19], [46], [54].

Supporting our hypothesis, results in Fig. 1 indicate significant protection against disintegration of the tubulointerstitial structures in PARP inhibitor treated transplanted kidneys. We found the studied aspects of the molecular background to be similar to the mechanism demonstrated in other oxidative stress models [16]–[18], [27]. PARP inhibition decreased the predominantly PARP-1 mediated [12] nuclear poly-ADP-ribosylation (Fig. 2a, B) and oxidative/nitrating stress seen as decreased nitrotyrosine staining in PARP inhibitor treated transplanted kidney samples (Fig. 2C, D). This is in accordance to our previous studies in various oxidative stress model systems [18], [32], [27], [55]. PARP inhibition also increased the expression of Bcl-2 and decreased the Bax expression in transplanted kidneys (Fig. 3A, B) when compared to controls. That is, suppressing the proapoptotic Bax and activation of the expression of anti-apoptotic Bcl-2 clearly promote survival and help to maintain tubulointerstitial structures as demonstrated here for the first time.

The mechanism of how PARP inhibitors modulate the expression of Bax and Bcl-2 is not clear. Only recent data indicate Bax expression is activated by p53 in oxidative stress [56]. Also, PARP-1 poly-ADP-ribosylates p53 and prevents its Crm1 dependent nuclear export [57]. Therefore, inhibition of PARP-1 may prevent poly-ADP-ribosylation of p53 initiating its Crm1 mediated export from the nucleus and so attenuates p53 dependent gene expressions. PARP inhibition modulates apoptotic and necrotic cell death by mitochondrial permeability transition and by AIF release [48], [49]. Our observations that PARP inhibitors enhance Bcl-2 and Bax ratio suggest this could be another pathway by which they promote cell survival. Furthermore, we demonstrated that t-Bid, a highly apoptotic protein, formed in large quantities in transplanted kidneys compared to control tissues. The PARP inhibitor significantly decreased this quantity (Fig. 3A, B) indicating a novel anti-apoptotic pathway for PARP inhibition. Although the importance of t-Bid in graft survival has not yet been studied, our data indicate a role for the molecule to promote cell death in transplanted kidneys.

Studying the signaling pathways in transplanted kidneys, we found PARP inhibitors activate the cytoprotective PI-3-K-Akt pathway (Fig. 5A, B), which contributes to graft survival and preservation of tubulointerstitial structures (Fig. 1A, [58]). PARP inhibitor induced Akt activation was first demonstrated by Veres et al. indicating a protective role in septic shock [17], [20]. PI-3-K-Akt pathway plays a significant role in the protection of mitochondrial membrane system and in the inhibition of cell death pathways during oxidative stress [46], [59]. Therefore, PARP inhibition induced activation of Akt may have significant role in kidney graft survival. The regulation of NF-kappaB is complex. Similarly to that of others [60], our data indicate a parallel behavior for Akt and NF-kappaB activation (Fig. 4). Thus, it is likely that Akt initiated NF-kappaB activation may contribute to the survival of transplanted kidneys under our experimental conditions. The effect of PARP inhibition on the MAP kinases was also determined. Our results indicate modest alterations in ERK1/2 and p38 MAP kinase pathways, while PARP inhibition significantly suppressed the JNK1/2 activation in transplanted kidneys (Fig. 6A, B). This may also have significance during rejection since JNK1/2 promotes mitochondrial permeability transition and cell death [61]. Our observations correspond with previous data showing PARP inhibitors suppress JNK activation in oxidative stress [25], [61]. Also, since JNK1 activation negatively affects graft survival [62]-[64], JNK1 inhibition may play a positive role in transplanted kidneys. We predict PARP inhibition possesses similar positive effects as JNK inhibitors by indirectly suppressing JNK1/2 in transplants. The mechanism, by which PARP inhibitors suppress JNK activation can be mediated by PARP inhibition induced MAP kinase phosphatase-1 activation as we demonstrated before [19]. Therefore, the disintegration of the tubulointerstitial structures in transplanted kidneys can be at least in part the consequence of JNK1/2 activation induced cell death. Moreover, since hypoxia activates JNK1/2 through the Ask1-MEKK4/7-JNK1/2 pathways, JNK1/2 activation in transplanted kidneys is most likely a consequence of ischemia-reperfusion, inflammatory cytokines and monocyte/macrophage induced oxidative stress.

In conclusion, rejection in transplanted kidneys during early and late phases is induced in a large extent by oxidative stress of diverse sources (e.g. ischemia-reperfusion, inflammatory cytokines and monocytes/macrophages). Inflammation or modulation of signaling cascades that lead to mitochondrial damages facilitate pro-apoptotic and necrotic processes and result in loss of nephrons or impairment of tubulo-interstitial structures. Our work demonstrates that, oxidative stress activates PARP, which initiates cell death and shifts kinase cascades by suppressing the cytoprotective PI-3-kinase-Akt pathway and by activation of JNK. The alterations promote mitochondria mediated cell death that significantly contributes to kidney graft rejection. The inhibition of PARP reverses these processes and shifts Bcl-2/Bax ratio into the cytoprotective direction. The protective mechanisms of PARP inhibitors are markedly different from that of the widely used immunosuppressive therapies. Our results suggest the supplementation of immunosuppression with PARP inhibitors could provide a novel way to prolong graft survival.
